# Indian Hedgehog (IHh) Protein and COX-2 as Biomarkers to Define the Mechanism of Epilepsy and Gastrointestinal Problems as Comorbid Medical Illnesses in Autism Spectrum Disorder: Combining ROC Curves to Improve Diagnostic Values

**DOI:** 10.3390/jcm13226695

**Published:** 2024-11-07

**Authors:** Afaf El-Ansary, Manan Alhakbany, Hanan A. Alfawaz, Laila Y. Al-Ayadhi

**Affiliations:** 1 Autism Center, Lotus Holistic Alternative Medical Center, P.O. Box 110281, Abu Dhabi 23251, United Arab Emirates; 2 Department of Physiology, Faculty of Medicine, King Saud University, Riyadh 11495, Saudi Arabia; malhakbany@gmail.com (M.A.); lyayadhi@ksu.edu.sa (L.Y.A.-A.); 3 Department of Food Science and Nutrition, College of Food and Agriculture Sciences, King Saud University, P.O. Box 22452, Riyadh 11495, Saudi Arabia; halfawaz@ksu.edu.sa

**Keywords:** Indian hedgehog (IHh) protein, COX-2, ASD, epilepsy, gastrointestinal problems, ROC curves, diagnostic biomarkers

## Abstract

**Background/Objectives**: Autism spectrum disorder (ASD) is a neurodevelopmental disorder that is increasing throughout the world. Nevertheless, no specific diagnostic or even risk assessment marker is available. Combining more than one marker can improve the diagnostic value of each marker alone and maximize the AUC for ROC curve. Hedgehog (Hh) signaling modulates both intestinal inflammation and immunity. Cyclooxygenase-2 (COX-2) is required for inflammation, and it has been related to epileptic seizures. COX-2 generates prostaglandins-EP2 (PGE2) in the brain, which plays a major role in neuropathology and epilepsy. This study aims to understand the pathophysiology of ASD by investigating the diagnostic value of COX-2 and IHh using independent and combined ROC curves. **Methods**: COX-2 and IHh were measured in 40 children with ASD and 41 age and sex-matched controls using ELISA. Statistical analyses were performed using ROC curves, odds ratios, and multiple logistic regression models. **Results**: Higher levels of COX-2 and IHh were observed in the plasma of patients with autism than in controls. The ROC curve of HIh and COX-2 independently showed poor and fair AUCs of 0.662 and 0.776, respectively, while the combined ROC for both variables in relation to the control group increased the AUC = 0.831 significantly (*p* < 0.001). **Conclusions**: Thus, combining these variables could be a useful diagnostic marker for ASD.

## 1. Introduction

With a frequency of approximately 2% in children in the United States, autism spectrum disorder is a neurodevelopmental disorder that is common throughout the world. According to estimates, there were 42,500 confirmed cases of ASD in Saudi Arabia, with many more going undiagnosed [1].

Nevertheless, no scientifically validated biological marker exists that can be used to offer a diagnosis or risk assessment for autism spectrum disease, or to suggest the best course of action.

Initial research on immunological, metabolic, genetic, and neuroimaging diagnostic biomarkers is promising, particularly when combined with other measures like behavioral evaluations. However, large validation studies and the use of suitable control populations are absent.

It seems that the field of biomarker research in autism spectrum disease is still in its infancy, despite its potential. When it comes to initiating therapies early, biomarkers by which to stratify autism spectrum disorder risk during the prenatal and postnatal pre-symptomatic period may be particularly helpful in starting interventions early, when they might be most effective, and biomarkers than can predict treatment response may expedite habilitation for those already diagnosed [2].

In disease screening, it is usual practice to collect many biomarkers from each participant. These biomarkers frequently reflect multiple features of the disease, such as severity, subtype, and so on. Any one of these markers alone may not be an effective diagnostic tool, but combining them may assist in attaining higher accuracy. The receiver operating characteristic (ROC) curve is an effective tool for determining diagnostic accuracy. Many biomarker combination approaches are based on maximizing the area under the ROC curve (AUC) [3].

The hedgehog (Hh) signaling pathway is essential for embryonic growth and patterning, as well as maintaining adult tissue homeostasis. Interestingly, Hh signaling modulates both intestinal inflammation and immunity. Lees et al. (2008) provided the first evidence that Hh signaling is required for the proper regulation of inflammatory responses in the mammalian gut, notably in inflammatory bowel disease (IBD) [4]. The Hh signaling pathway interacts with many signaling pathways, including Wnt and NF-κB signaling, which govern mammalian intestinal homeostasis and play essential roles in IBD [5,6,7].

Cyclooxygenase-2 (COX-2) is required for inflammation, which is caused by a variety of stimuli such as cytokines, endotoxins, and growth factors. It also triggers the production of prostaglandins, which lead to the formation of inflammation symptoms [8,9,10] and the degradation of tissue integrity [11].

Preclinical and clinical studies are beginning to provide evidence that neuronal hyperexcitability in epilepsy with varying etiologies is inherently linked to central nervous system inflammation. COX-2 has been connected through an inflammatory signaling mechanism to epileptic seizures. One of the main prostanoids generated in the brain by COX-2 is prostaglandins-EP2 (PGE2), and it plays a major role in neuropathology. Thus, PGE2 and BDNF levels in the hippocampal regions—which are linked to epilepsy—were lowered by inhibiting COX [12,13].

Comorbidities in ASD may have atypical presentations and symptoms, making diagnosis more difficult [14]. Despite these possible challenges in diagnosis, the reported comorbidity load in ASD remains high; in a population-based study in Sweden, Lundström et al. [15] discovered that more than half of patients with ASD had four or more comorbid illnesses. Given that certain health conditions are more common in people with ASD [15], there may be a shared etiology between these numerous comorbidities and ASD [16,17,18,19].

Compared to 2% to 3% of the general population, 25% to 40% of ASD patients have epilepsy, and seizures significantly worry their families. Researchers found that patients with ASD are more likely to acquire epilepsy if they have an intellectual disability, an underlying neurologic illness, a family history of epilepsy, or a significant cognitive delay.

The most prevalent GI disorders documented in autism are persistent constipation, stomach pain with or without diarrhea, and constipation-induced encopresis [20]. Furthermore, greater autistic symptom severity is related with an increased risk of having GI issues [21,22].

The goal of this study is to better understand the pathophysiology of ASD by investigating the diagnostic value of COX-2 and IHh in the blood plasma of people with the disorder. This will be accomplished by using independent and combined ROC curves. The importance of both variables in the development of epilepsy and GI disorders as prevalent comorbidities in ASD will be emphasized.

## 2. Materials and Methods

The ethics committee of the medical college at King Saud University approved the study procedure in compliance with the most recent Declaration of Helsinki. This study included 40 autistic patients and 42 age- and gender-matched controls. The assumption behind selecting controls and cases is that they are from the same source population. The little discrepancy in numbers between the two groups resulted from a lack of blood samples taken from two cases. All subjects gave written informed consent through their parents and agreed to take part in the study. The study participants were enrolled in the Autism Research and Treatment Center (ART Center) clinic. The ART Center clinic’s sample population included children with ASD. All study participants’ ASD diagnoses were confirmed via the Autism Diagnostic Interview—Revised (ADI-R), the Autism Diagnostic Observation Schedule (ADOS), and the 3DI (Developmental, Dimensional, and Diagnostic Interview) protocols.

This study included autistic children aged 2 to 12 years old. All tested negative for the fragile X gene. The control group was recruited from the pediatric clinic at King Saud Medical City, and their average age ranged from 2 to 14 years. Dysmorphic characteristics, fragile X syndrome, and other neurological problems such as seizures, bipolar disorder, or any known medical issues were all excluded. During a parental interview, all participants were asked if they had ever had a physical illness. All patients and controls in the research followed comparable but not identical diets, and none were on a particular gluten-free diet. This study did not include children with known endocrine, cardiovascular, lung, liver, renal, or other medical disorders. All of the patients and controls in the study ate a comparable but not identical diet, and no one followed a special high-fat or fat-restricted diet.

### 2.1. Blood Sampling

After the overnight fasting period, participants had their blood drawn into 3 mL EDTA-filled blood collection tubes by a skilled technician. Blood was drawn right away, and it was centrifuged for 20 min at 4 °C at 3000× *g* using A Boeco centrifuge (Model U-320R, Boeco, Hamburg, Germany). After being separated, the plasma was kept at −80 °C until it was needed. It was divided into three 0.5 mL aliquots to prevent repeated freeze–thaw cycles.

### 2.2. Biochemical Assays

#### 2.2.1. Cyclooxygenase-2 (COX-2)

To assess the amounts of COX-2, a quantitative sandwich enzyme-linked immunosorbent assay (ELISA) kit from CUSABIO (8400 Baltimore Avenue, Room 332, College Park, MD, USA) was utilized. Following the manufacturer’s recommendations, the measurement was performed with a minimum detectable dosage of 0.31 ng/mL.

#### 2.2.2. Indian Hedgehog (IHh) Protein

Plasma levels of IHh were measured using a commercially available sandwich enzyme immunoassay (ELISA) kit from Cusabio Biotech Co., Ltd. (Wuhan, China).

#### 2.2.3. Statistical Analysis

The data were analyzed using IBM SPSS software version 22.0 (IBM Inc., Armonk, NY, USA). The Shapiro–Wilk test was used to determine data normality in each group. The results were shown as the minimum, maximum, and median. The Mann–Whitney test was used to compare two non-parametric groups, with *p*-values ≤ 0.05 indicating significant differences. The Spearman rank correlation coefficient (R) was used to link different non-parametric variables. The Enter method was used to conduct a logistic regression analysis for the patient group, with one variable serving as the dependent variable and the second as the independent.

The link between the biomarkers and clinical state is displayed in the combined receiver operatic characteristic (ROC) curves by the odd ratios (ORs) derived from the logistic regression study. For every logistic regression model, ROC curves were produced. A non-parametric technique was used to calculate the area under the curve (AUC) for each marker combination. In logistic regression, odds ratios greater than 1 indicate “positive effects” because they increase the likelihood. Those between 0 and 1 are known as “negative effects” since they tend to reduce probabilities. A ratio of odds equal to 1 indicates “no association”. A ratio of odds cannot be less than 0.

When assessing the predictive ability of biomarkers, the area under the curve (AUC) is a helpful measure. The AUC is a useful metric for assessing the predictive ability of biomarkers. While a curve at the diagonal (AUC = 0.5) lacks diagnostic relevance, a highly excellent predictive marker is shown by an AUC value near 1.00. When the AUC value is close to 1.00, there is always a biomarker with the right specificity and sensitivity values [2]. When considering potential biomarkers for ASD, high sensitivity indicates that the majority of patients will be diagnosed with ASD; high specificity, on the other hand, indicates that healthy persons will rarely test positive for the variable under investigation. Furthermore, adding two additional markers to an ROC curve analysis often increases their specificity [2], implying that employing a panel of factors rather than a single variable may be highly useful as a diagnostic tool.

## 3. Results

**Figure 1:**
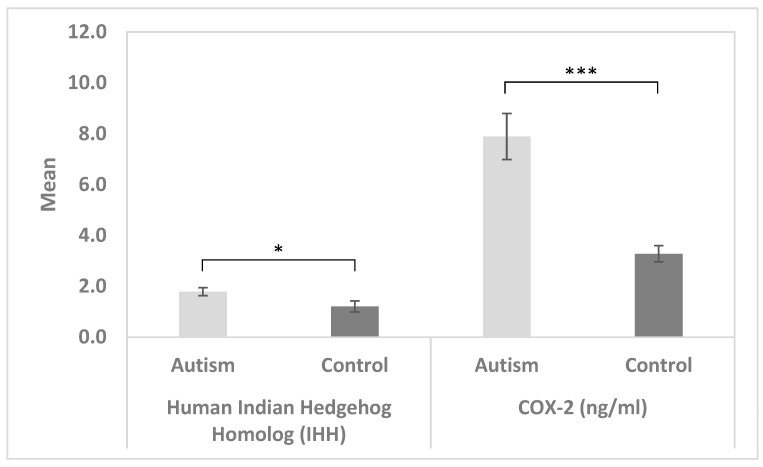
Plasma levels of the IHh protein and COX-2 in controls and autistic children. There were statistically significant IHh and COX-2 plasma levels in autism compared to controls (* *p* = 0.048 and *** *p* = 0.001, respectively).

Table 1 demonstrates the available demographic data of autistic and control participants recruited in the study. Table 2 and Figure 1 both describe the comparison between the control group and the patient group for IHh and COX 2 using the Mann–Whitney test (non-parametric data). Both parameters’ plasma levels were significantly higher in individuals with ASD compared to healthy controls (*p* < 0.005).

**Figure 2:**
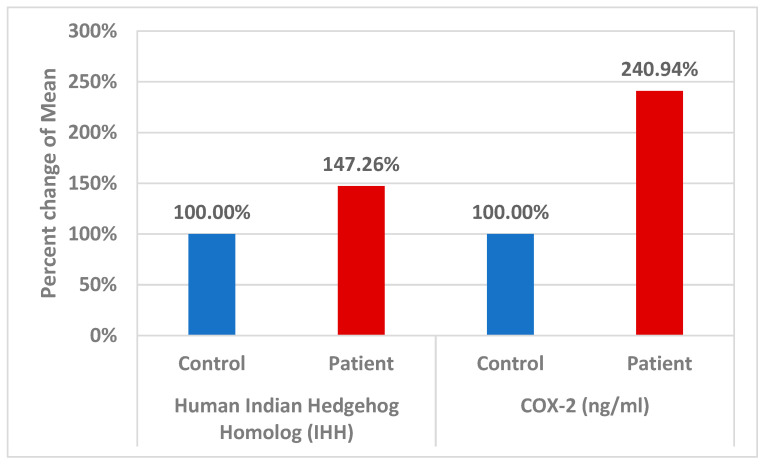
Percentage change in mean for plasma IHh and COX-2 in the patient group relative to healthy controls (presented as 100%).

The percentage changes in the levels of the two measured variables, IHh and COX-2, were significantly higher in individuals with ASD (at 147 and 240%, respectively) compared to healthy controls (*p* < 0.001) (Figure 2).

Table 3 shows that there is a positive correlation between IHh and COX-2 levels using Spearman correlation (*p* = 0807), but it is a weak association (R = 0.34).

**Figure 3:**
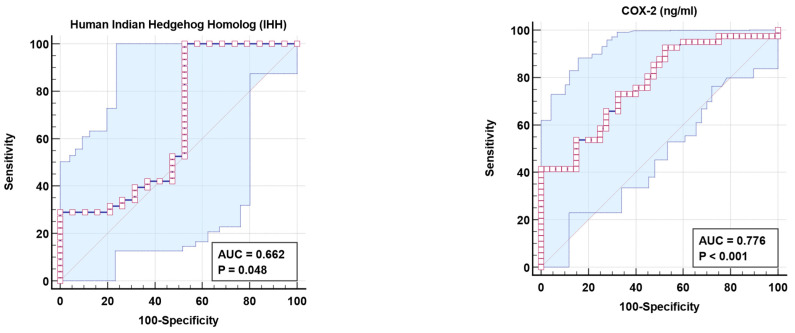
ROC curve for plasma IHh and COX-2 of patient group according to the control group.

An ROC curve analysis was performed to evaluate the utility of HIh and COX-2 in the early diagnosis of ASD. Figure 3 and Table 4 show the AUCs, cutoff values, specificity, and sensitivity of the two measured parameters independently. Both HIh and COX-2 independently showed poor and fair AUCs of 0.662 and 0.776, respectively.

Table 5 describes the logistic regression test results for the patient group as a dependent variable, with (human Indian hedgehog homolog (IHh) and COX-2 (ng/mL)) as independent variables using the Enter method.

**Figure 4:**
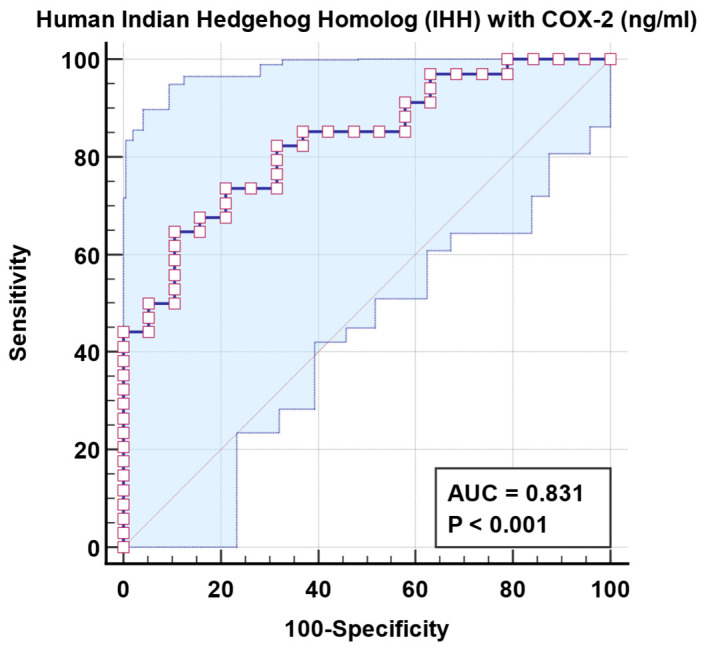
Combined ROC for plasma IHh and COX-2 of patient group according to the control group.

Figure 4 and Table 6 show that the combined ROC for plasma IHh and COX-2 in relation to the control group increased the AUC = 0.831 significantly (*p* < 0.001).

## 4. Discussion

In the present study, higher levels of both COX-2 and IHh were observed in the plasma from patients with autism than in controls (Table 2). The high significant increase in COX-2 is evidence for inflammation playing a significant role in ASD. Cyclooxygenase-2 (COX-2) converts arachidonic acid into prostaglandin H2, which promotes acute and chronic inflammation. This can find support in the study of Yoo et al. [23], which proves that polymorphism of the COX-2 gene can contribute to the genetic predisposition to AS. Interestingly, El-Ansary et al. [24] reported that increased levels of both COX-2 and PGE2 have been discovered in the plasma samples of autistic people. Increased plasma PGE2 levels were concurrent with significantly reduced levels of α-synuclein, which plays a vital role in synaptic processes, including synaptic pool preservation, vesicular stabilization, and synaptic plasticity [24]. In relation to glutamate excitotoxicity and mitochondrial dysfunction as ASD etiological mechanisms, the ROC combination of α-Syn, COX-2, and PGE2/its EP2 receptor may be a predictive diagnostic biomarker of ASD, suggesting that the above-mentioned diagnostic markers might aid in early intervention [24].

The significantly increased level of IHh in individuals with ASD compared to healthy controls presented in Table 1 is in good agreement with the work of Bashir and AL-Ayadhi [25], which points to aberrant IHh protein levels in ASD as compared to age-matched controls. This could be related to gastrointestinal problems as a co-morbidity in ASD. Wnt and hedgehog signaling interact at the transcriptional level [26]. During chronic intestinal inflammation, epithelial IHh promotes the production of Wnt2B in intestinal mesenchymal cells [27]. Wnt signaling can also interact with cytokine signaling and nuclear factor kappa (NF-κB) in many ways to stimulate inflammatory responses [28]. Additionally, abnormalities in the CNS, GI, and nociception have been found in ASD patients [29,30,31], and growing evidence suggests that these abnormalities are linked to changes in the COX-2/PGE2 pro-inflammatory pathway.

Evidence indicating the altered expression of several cytokines, chemokines, and other immune-related molecules in epilepsy suggests inflammation, being a key component in epilepsy pathophysiology, as a common co-morbidity in ASD. In addition to cytokines, after hippocampal kindling in rats, activation of the proinflammatory enzyme COX-2 was seen in the hippocampus and neocortical neurons. This shows that COX-2 upregulation and its products, PGE2, are essential signaling events in epileptogenesis, which further aggravates seizure severity [32,33,34].

Table 4 and Figure 3 demonstrate that independent COX-2 and IHh proteins exhibit poor predictive diagnostic properties in patients with ASD. Low AUC results of 0.662 and 0.776 were insufficient to make any recommendations regarding discrimination against people with ASD and healthy controls. Nonetheless, the combined ROC of the two variables was successful in increasing the AUC to 0.83 while maintaining adequate specificity and sensitivity and delivering good diagnostic value. The combination of the two variables reveals that both signals play a role in the clinical presentation of ASD as a whole, as well as the most common co-morbidities: GI difficulties and seizures.

COX-2 is exclusively localized to excitatory neurons, such as glutamatergic cells [35]. In an attempt to clarify the combining effects of increased IHh and COX-2 in the etiology of ASD, it is interesting to consider the work of Strauss and Marini [36], which reported that glutamate excitotoxicity enhanced COX2 mRNA in cerebellar granule neurons and promoted de novo prostaglandin generation. The authors hypothesized that the glutamate-mediated induction of COX-2 contributes to excitotoxic and apoptotic cell death. Excitotoxicity as an ascertained etiology in ASD also affects the mitochondria function since excessive glutamate disrupts Ca^+2^ balance and ATP production and further leads to the formation of reactive oxygen species. It is also associated with endoplasmic reticulum stress due to the pathological Ca^+2^ signal evoked by excess glutamate [36].

In relation to glutamate excitotoxicity, hedgehog signaling is crucial for neuronal circuit construction and synaptic plasticity in the postnatal rodent hippocampus. It regulates GABAergic transmission, intracellular Ca^2+^ signaling, and the BDNF-TrkB signaling pathway [37,38]. There is considerable evidence that epilepsy is caused by an inherent or acquired impairment in GABAergic activities [39]. Blocking GABAergic inhibition in the healthy brain causes abrupt epileptic discharges.

Moreover, some researchers have found that hedgehog signaling pathway activation leads to an etiology of ASD [40]. The hedgehog pathway is linked to a number of developmental and cell-survival pathways in the brain. Pathway activation can occur through both canonical (patched 1) and non-canonical methods. Several kinases, including phosphokinase (PKA), glycogen synthase kinase 3β (GSK3-3β), ribosomal protein S6 kinase (S6K), and dual-specificity tyrosine phosphorylation regulated kinase 1B (DYRK1B) can regulate Gli1/2 phosphorylase activity [41]. DYRK1B and S6K are linked to the mTOR/Akt signaling pathway. DYRK1B controls mTOR/Akt, whereas mTORC1 phosphorylates S6K. As a result, it appears that Gli1/2 is linked to phosphoinositide 3-kinase (P13K) mTOR/Akt. Gli is a crucial factor in dual regulation. Thus, if there is an over-activation of mTOR signaling due to upstream activity, increased S6K activity leads to the upregulation of hedgehog signaling in the non-canonical pathway. Upregulation of the PI3K-AKT/mTOR signaling pathway involves many human brain abnormalities, including autism, and it is a suggested target to treat ASD [42].

Epilepsy causes unpredictable seizures as a result of aberrant electrical activity. Excitotoxicity is one of the primary causes of these seizures, as seizure activity is predominantly conveyed from one neuron to the next via excitatory glutamatergic transmission. Glutamate-induced excitotoxicity is known to cause neuronal death in epilepsy, and elevated glutamate levels have been seen in epileptic human brain tissues as well as animal models of epilepsy [43,44,45]. COX-2 is uniformly expressed in neurons in the cortex and hippocampus after a seizure, indicating a link between neuronal activity and COX-2 expression [35,46].

The inter-relationship between the two variables, together with their involvement in epilepsy and GI problems in individuals with ASD, can be discussed through the logistic regression presented in Table 5. In logistic regression, “positive effects” are defined as odds ratios greater than 1, suggesting that they increase probability. The positive correlation and odds ratio values of 2.258 and 1.524 for IHh and COX-2, respectively, could support their role in the pathophysiology of ASD.

**Figure 5:**
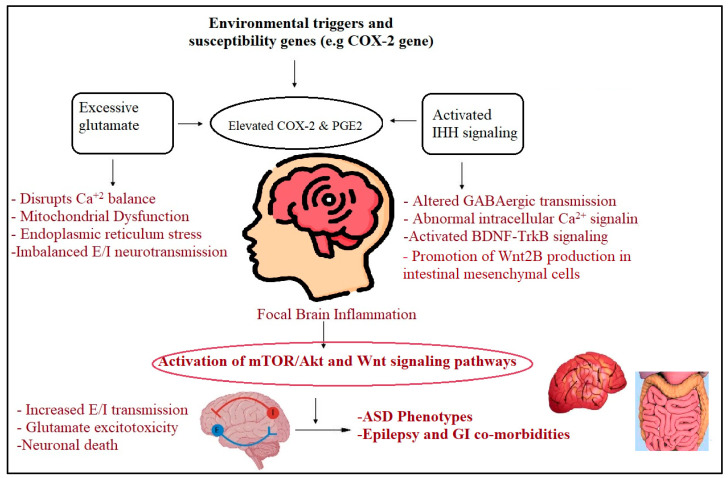
Illustration of the relationship between COX-2 and IHh in inducing glutamate excitotoxicity and neuroinflammation as etiological mechanisms of ASD. The contributions of both variables in epilepsy and GI problems, both major co-morbidities in ASD, are also presented.

Figure 5 demonstrates how COX-2 and IHh work together to produce glutamate excitotoxicity and neuroinflammation, two etiological processes of ASD. Additionally, it is demonstrated that seizures and gastrointestinal disorders are important co-morbidities of ASD.

## 5. Conclusions

Since epilepsy and gastrointestinal problems are significant medical complications that arise in people with autism, seizures may initially start in adolescence or maturity. Early intervention to prevent or reduce the frequency of seizures may benefit from the identification of potential biomarkers that could predict the likelihood of epilepsy development in autism. The significant increase in predictive diagnostic value of combining the two studied ASD biomarkers, COX-2 and IHh, as well as our understanding of their roles in glutamate excitotoxicity and neuroinflammation as two confirmed etiological mechanisms of ASD, suggests that these variables may be useful diagnostic markers for ASD in addition to epilepsy and gastrointestinal problems, which are common comorbidities. This could help with early intervention.

### Limitations

The disadvantages of the current study are the limited sample size and changes in the number of evaluated samples caused by insufficient samples.

## Figures and Tables

**Table 1:** Demographic available data of autistic and control participants.

Variable	Autism (40)	Control (42)
Age	2–12 Years	2–14 Years
Sex	All males	All males
**GIT**		
Colic	11	1
Constipation	12	2
Diarrhea	2	0
**Birth complications**		
CS/forceps delivery	19	3
Autoimmunity family history	23	0
**Allergy**		
Food allergy	13	0
Eye allergy	12	1
Skin allergy	14	2
**Parental age**		
Average age of father (years)	31.5 (±6.7)	30.8 (±5.3)
Average age of mother (years)	35.5 (±7.2)	34 (±8.1)
**Epilepsy**		
	0	0

**Table 2:** Comparison between the control group and the patient group.

Parameters	Groups	N	Min.	Max.	Mean ± S.D.	Median	*p*-Value
Human Indian Hedgehog Homolog (IHh)	Control	19	0.070	2.570	1.21 ± 0.94	1.398	0.048
	Patient	38	0.760	3.790	1.79 ± 0.97	1.426	
COX-2 (ng/mL)	Control	40	0.910	8.640	3.28 ± 2.00	2.501	0.001
	Patient	41	0.050	20.570	7.89 ± 5.80	4.995	

**Table 3:** Correlations between plasma IHh and COX-2 using Spearman correlation.

Parameters	R (Correlation Coefficient)	*p*-Value	
Human Indian hedgehog homolog (IHh) with COX-2 (ng/mL)	0.034	0.807	P ^a^

^a^ Positive correlation.

**Table 4:** ROC results for patient group according to the control group as a reference group.

Parameters	AUC	Cut-Off Value	Sensitivity %	Specificity %	*p*-Value	95% CI
Human Indian Hedgehog Homolog (IHH)	0.662	0.647	100.0%	47.4%	0.048	0.498–0.827
COX-2 (ng/mL)	0.776	8.784	41.5%	100.0%	0.001	0.676–0.876

**Table 5:** Logistic regression (patient group).

Parameters	Regression Coefficient	Standard Error	Odds Ratio	95% CI for Odds Ratio		*p*-Value
				Lower	Upper	
Human Indian Hedgehog Homolog (IHh)	0.815	0.380	2.258	1.072	4.760	0.032
COX-2 (ng/mL)	0.422	0.174	1.524	1.084	2.144	0.015

**Table 6:** Combined ROC results of the following parameters for the patient group, using the control group as a reference group.

Parameters	AUC	Sensitivity %	Specificity %	*p*-Value	95% CI
Human Indian Hedgehog Homolog (IHh) with COX-2 (ng/mL)	0.831	64.7%	89.5%	0.001	0.723–0.939

## Data Availability

The original contributions presented in thus study are included in the article. Further inquiries can be directed to the corresponding author.
